# A Clinicopathologic, Molecular, and Prognostic Comparison Between Early- and Late-Onset Colorectal Cancer in Korea: A Single-Center Retrospective Cohort Study

**DOI:** 10.3390/jcm15051736

**Published:** 2026-02-25

**Authors:** Sung Bin Park, Hoon Sup Koo, Dae Sung Kim, Jieun Ryu, Jieun Shin, Jun Suk Oh, Kyu Chan Huh

**Affiliations:** 1 Division of Gastroenterology, Department of Internal Medicine, Konyang University Hospital, Daejeon 35365, Republic of Korea; mirojang@naver.com (S.B.P.); hornkim@kyuh.ac.kr (D.S.K.); 2 Department of Gastroenterology, Myunggok Medical Research Institute, Konyang University, Daejeon 35365, Republic of Korea; koo7574@kyuh.ac.kr; 3 Department of Gastroenterology, Healthcare Center, Konyang University Hospital, Daejeon 35365, Republic of Korea; 200721@kyuh.ac.kr; 4 Department of Biomedical Informatics, College of Medicine, Konyang University, Daejeon 35365, Republic of Korea; 910037@kyuh.ac.kr; 5 Department of Pediatrics, Konyang University Hospital, Daejeon 35365, Republic of Korea; joojang87@kyuh.ac.kr

**Keywords:** early-onset colorectal cancer, late-onset colorectal cancer, clinicopathologic and molecular characteristics, prognosis

## Abstract

**Background/Objectives**: The incidence of early-onset colorectal cancer (EO-CRC), diagnosed before age 50, is increasing worldwide; however, comparative data between patients with EO-CRC and late-onset colorectal cancer (LO-CRC) in Asian populations remain limited. We compared the clinicopathological, molecular, and prognostic characteristics of EO-CRC and LO-CRC in a tertiary-center cohort. **Methods**: This retrospective cohort study included patients with histologically confirmed colorectal adenocarcinoma treated at a single tertiary referral center between January 2011 and December 2024. Patients were classified as having EO-CRC (<50 years) or LO-CRC (≥50 years). Demographic and lifestyle factors, clinicopathological characteristics, laboratory findings including blood tests and tumor markers, and molecular profiles such as microsatellite instability (MSI) status and selected gene mutations were compared. Overall survival and associated prognostic factors were evaluated using multivariate analysis. **Results**: Among 1383 patients, 104 had EO-CRC and 1279 had LO-CRC. Patients with EO-CRC reported smoking and alcohol consumption more frequently, had fewer comorbidities, and showed a higher prevalence of distal tumors, particularly rectal cancer, with a lower T stage. Nodal and distant metastatic stages were comparable between the groups, with no difference in the proportion of stage IV disease. Laboratory parameters, tumor marker levels, MSI status, and other available molecular markers were not significantly different. Overall survival did not differ significantly between EO-CRC and LO-CRC. **Conclusions**: EO-CRC exhibited distinct clinical features; however, molecular characteristics and survival outcomes were similar to those of LO-CRC. Prognosis is primarily determined by disease stage rather than the age at diagnosis, supporting the importance of early detection strategies in high-risk populations.

## 1. Introduction

Colorectal cancer (CRC) is a leading cause of cancer-related morbidity and mortality worldwide [1]. Traditionally considered as a disease of older adults, CRC screening primarily targets individuals aged ≥50 years in many countries across both Western and Asian regions, including several East Asian countries [2]. However, epidemiological trends in recent decades have shown a sustained increase in the incidence of early-onset colorectal cancer (EO-CRC), defined as CRC diagnosed before age 50 [3,4]. In the United States, EO-CRC is projected to account for approximately 11% of colon cancers and 23% of rectal cancers by 2030 [5,6]. This growing public health burden has prompted a re-evaluation of screening guidelines. Consequently, several countries—including the United States—have lowered the recommended age for initiating screening from 50 to 45 years [7,8,9]. Beyond epidemiologic trends, EO-CRC has also been characterized by distinct clinicopathologic features. Compared with late-onset colorectal cancer (LO-CRC), EO-CRC has been reported to present at more advanced stages, with more frequent aggressive histopathological characteristics, such as mucinous or signet ring cell histology, poor differentiation, and perineural or vascular invasion [10,11,12]. However, the effect of these clinicopathologic differences on clinical outcomes remains unclear. Although a subset of EO-CRC cases are associated with hereditary syndromes, such as Lynch syndrome, most cases arise sporadically, highlighting the need to understand age-specific environmental and molecular pathogenesis [13].

Interest in EO-CRC has increased in Korea, a country with a high CRC incidence. According to recent data from the Korea Central Cancer Registry, EO-CRC incidence increased rapidly until around 2011, but has since plateaued [14]. This pattern differs from the continuously rising trends reported in many Western countries, suggesting that EO-CRC may follow epidemiological trajectories distinct from those observed in Western populations and highlighting the need for population-specific investigations.

Nevertheless, studies comprehensively describing the clinical, pathological, and molecular genetic characteristics of EO-CRC in Asian cohorts remain limited. In addition, data linking these characteristics to patient outcomes in region-specific populations are scarce. For example, a recent Korean study reported that in contrast to global patterns, a considerable proportion of EO-CRC cases were diagnosed at early T stages [15]. Such discrepancies underscore the need for in-depth characterization of patients with EO-CRC across diverse clinical settings.

In this context, we characterized the clinicopathological and molecular genetic features of EO-CRC in a large tertiary-center cohort and evaluated clinical outcomes according to age at diagnosis. These findings may provide evidence to inform strategies for early detection and risk stratification of CRC in individuals aged <50 years and support the development of personalized management strategies.

## 2. Materials and Methods

This retrospective, observational cohort study was conducted at a single tertiary university hospital serving as a regional referral center, without an independent external cohort for validation. The study was approved by the hospital’s Institutional Review Board (IRB) (IRB No. 2025-03-020, approved on 17 April 2025), and patient data were obtained from electronic medical records. Given the minimal-risk retrospective design, the requirement for informed consent was waived by the IRB, and all analyses were performed on de-identified data. The study period was from 1 January 2011 to 31 December 2024. The start year was selected to align with reported changes in EO-CRC incidence trends in population-based registry data [14].

We included patients with histologically confirmed colorectal adenocarcinoma diagnosed at our institution. Patients were classified according to their age at diagnosis as having EO-CRC (<50 years) or LO-CRC (≥50 years). The eligibility criteria required that patients had received at least one treatment modality at our hospital—such as surgery, chemotherapy, or outpatient follow-up—after pathological confirmation of colorectal adenocarcinoma.

Patients were excluded if (1) medical records were insufficient to verify key clinical variables including date of diagnosis, stage, or major pathologic findings; (2) the final pathologic diagnosis was a non-adenocarcinoma tumor (such as gastrointestinal stromal tumor or neuroendocrine tumor); or (3) a known hereditary CRC syndrome or a clear predisposing condition for CRC was present. Known hereditary CRC was defined as a documented diagnosis of Lynch syndrome or familial adenomatous polyposis (FAP) in the medical record, supported by germline genetic testing results and/or genetics consultation notes, where available. Predisposing conditions included inflammatory bowel disease (ulcerative colitis or Crohn’s disease). Family history alone was not used to define hereditary CRC in this study.

**Figure 1:**
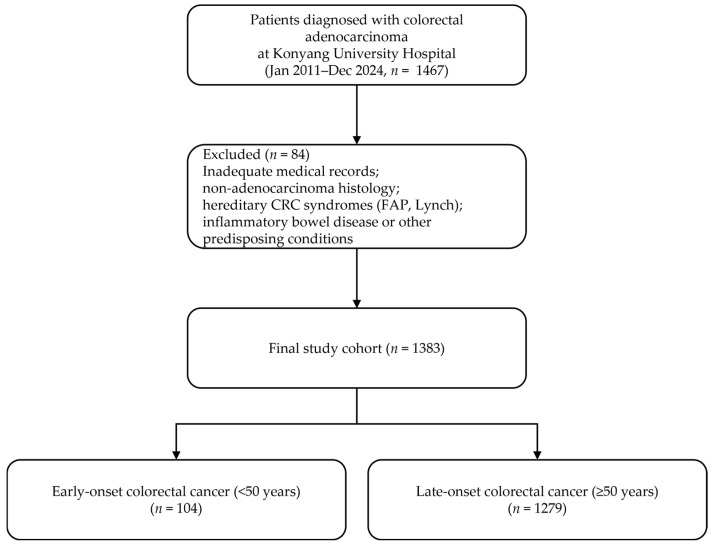
Flow diagram of patient selection for the study cohort.

The patients were identified through a systematic review of their electronic medical records. The final analytic cohort included 104 patients with EO-CRC and 1279 patients with LO-CRC (Figure 1).

Comparisons between the EO-CRC and LO-CRC groups focused on differences in demographic and lifestyle profiles, clinicopathological and laboratory features, molecular characteristics, and survival outcomes. All clinical and pathological data were retrospectively extracted from electronic medical records using a predefined case report form.

Demographic and lifestyle variables included sex, age at diagnosis, anthropometric measures (such as height, weight, and body mass index), smoking and alcohol consumption status, and major comorbidities (hypertension, diabetes mellitus, dyslipidemia, cardiovascular disease, and cerebrovascular disease). Smoking and alcohol consumption were categorized as never, former, or current based on medical records at the time of CRC diagnosis.

Clinical variables included the presence of major symptoms at diagnosis (such as hematochezia or melena, abdominal pain, changes in bowel habits, and weight loss). Laboratory variables included hemoglobin (Hb), white blood cell count (WBC), platelet count (PLT), aspartate aminotransferase (AST), alanine aminotransferase (ALT), total bilirubin, and serum creatinine. Tumor markers included carcinoembryonic antigen (CEA) and carbohydrate antigen 19-9 (CA19-9).

Tumor and pathological characteristics included tumor location (including rectum, left colon, and right colon), histological grade (including well/moderate/poorly differentiated), histological subtype (including adenocarcinoma, mucinous carcinoma, and signet ring cell carcinoma), depth of invasion (such as submucosa, proper muscle, and serosa), lymphatic/vascular/perineural invasion, lymph node metastasis, and distant metastasis. Pathological staging was performed using the American Joint Committee on Cancer (AJCC) 8th edition of the tumor–node–metastasis (TNM) staging system [16].

Treatment variables included surgery, chemotherapy, and radiotherapy, along with the corresponding dates obtained from electronic medical records. Treatment modality was classified into eight categories according to treatment combinations: no recorded oncologic treatment, surgery only, surgery plus chemotherapy, surgery plus chemotherapy plus radiotherapy, surgery plus radiotherapy (no chemotherapy), chemotherapy only, chemotherapy plus radiotherapy (no surgery), and radiotherapy only. Among patients who underwent surgery, perioperative treatment timing was further categorized as surgery only, adjuvant-only therapy, or any neoadjuvant therapy based on the initiation date of chemotherapy and/or radiotherapy relative to surgery; neoadjuvant therapy was defined as treatment initiated before surgery, and adjuvant therapy as treatment initiated after surgery.

Molecular characteristics included microsatellite instability (MSI) status (microsatellite stable (MSS), MSI-low (MSI-L), and MSI-high (MSI-H)) and mutations in KRAS, NRAS, BRAF, TP53, and APC. Molecular test results were obtained from pathology and molecular diagnostic reports in the electronic medical record. Throughout the study period, molecular testing was performed as part of routine clinical practice according to test availability and clinical indication; therefore, not all patients underwent uniform molecular evaluation. MSI status was assessed using PCR-based microsatellite analysis. KRAS, NRAS and BRAF mutations were evaluated using PCR-based assays and, in selected cases, targeted next-generation sequencing (NGS). TP53 and APC mutation status was available only in a subset of patients who underwent targeted NGS. For each molecular marker, analyses were restricted to patients with available test results, and missing data were not imputed. The distribution of molecular testing according to age group and calendar period is summarized in Appendix A (Table A1).

For survival analyses, the primary endpoint was overall survival (OS), and the secondary endpoints were disease-specific survival (DSS) and disease-free survival (DFS). OS was defined as the time from diagnosis to death from any cause or the last follow-up. For DSS, deaths due to CRC were counted as events, whereas deaths from other causes were censored at the time of death. DFS was defined as the time from diagnosis to recurrence, and patients without documented recurrence were censored at the last follow-up.

Continuous variables are presented as mean ± standard deviation and were compared using Student’s *t*-test. Categorical variables are presented as frequencies and percentages and were compared using the chi-square test or Fisher’s exact test, as appropriate. Survival curves were estimated using the Kaplan–Meier method and compared with the log-rank test. Univariate Cox proportional hazards regression was used to assess the association between age group and survival outcomes (OS, DSS, and DFS). The multivariable Cox model for OS included age group and clinically relevant covariates, including treatment modality and diagnosis period. Treatment was categorized as surgery alone, any neoadjuvant therapy, adjuvant-only therapy, chemotherapy only, radiotherapy without surgery, or no recorded oncologic treatment. Diagnosis period was grouped as 2011–2014, 2015–2018, and 2019–2024. Prespecified sensitivity analyses were conducted by excluding rectal cancer cases and stratifying the cohort according to AJCC stage and diagnosis period. Hazard ratios (HRs) with 95% confidence intervals (CIs) are reported. All tests were two-sided, and *p* < 0.05 was considered statistically significant. Statistical analyses were performed using IBM SPSS Statistics version 27.0 (IBM Corp., Armonk, NY, USA).

## 3. Results

### 3.1. Demographic and Lifestyle Characteristics

The baseline demographic and lifestyle characteristics are summarized in Table 1. The mean age at diagnosis was 43.30 ± 5.64 years in the EO-CRC group and 69.99 ± 10.86 years in the LO-CRC group. Height and weight were significantly higher in patients with EO-CRC than in those with LO-CRC (164.89 ± 7.73 cm vs. 160.37 ± 9.45 cm, *p* < 0.001; and 64.64 ± 13.24 kg vs. 60.07 ± 11.87 kg, *p* = 0.001), whereas body mass index did not differ significantly between groups (23.67 ± 3.91 vs. 23.26 ± 3.62 kg/m^2^, *p* = 0.315).

Smoking status differed significantly between both groups (*p* < 0.001), with a higher proportion of current smokers in the EO-CRC group than in the LO-CRC group (36.5% vs. 18.7%). Alcohol consumption also differed (*p* = 0.002), with current drinking more frequent in the EO-CRC group (48.1% vs. 31.2%). Comorbidities were more common in LO-CRC (64.6% vs. 14.4%, *p* < 0.001). Sex distribution and the presence of symptoms at diagnosis did not differ significantly between groups (*p* = 0.084 and *p* = 0.059, respectively).

### 3.2. Laboratory Findings and Tumor Markers

The laboratory findings (including Hb, WBC, ALB, AST, and ALT) and tumor markers (CEA and CA19-9) at diagnosis are summarized in Table 2. Serum albumin was significantly higher in EO-CRC than in LO-CRC (3.98 ± 0.62 vs. 3.84 ± 0.56; *p* = 0.013). CEA and CA19-9 levels did not differ significantly between the groups (*p* = 0.286 and 0.964, respectively). No other laboratory parameters showed significant between-group differences (all *p* > 0.05). The number of patients analyzed varied by laboratory parameter (Hb, *n* = 1378; WBC, *n* = 1377; ALB, *n* = 1362; AST, *n* = 1371; ALT, *n* = 1371; CEA, *n* = 1241; CA19-9, *n* = 1137).

### 3.3. Clinicopathologic Characteristics

Clinicopathological characteristics are presented in Table 3. Tumor location differed significantly between the groups (*p* < 0.001). Distal tumors (rectum and left colon) were more common in patients with EO-CRC than in those with LO-CRC (82.7% vs. 73.8%), and the rectum was the most common primary site in EO-CRC group. In contrast, right-sided colon cancer was more frequent in LO-CRC.

Histological grade and subtype did not differ significantly between the groups (*p* = 0.522 and 0.101, respectively). Moderately differentiated adenocarcinoma was the most common subtype in both groups.

The depth of invasion differed significantly between the groups (*p* < 0.001). Tumors confined to the submucosa were more frequent in patients with EO-CRC than in those with LO-CRC (31.9% vs. 19.8%), whereas invasion beyond the serosa was more common in LO-CRC (72.6% vs. 44.9%). Lymphatic invasion, vascular invasion, perineural invasion, lymph node metastasis, and distant metastasis did not differ significantly between groups (all *p* > 0.05).

The distribution of T stages differed significantly between the groups (*p* = 0.001), with a higher proportion of T1–2 tumors in the EO-CRC group than in the LO-CRC group (32.6% vs. 18.0%). In contrast, N and M stages did not differ significantly (*p* = 1.000 and *p* = 0.625, respectively). Although the overall AJCC stage distribution differed (*p* < 0.001), reflecting a higher proportion of early stage (stages I–II) disease in EO-CRC, the proportion of stage IV disease was similar between groups. Stratification by M stage showed that the earlier T-stage distribution in EO-CRC was driven by M0 disease, whereas metastatic tumors were predominantly T4 in both age groups (Appendix A Table A2).

### 3.4. Treatment Characteristics

Treatment modalities are summarized in Table 4. The overall distributions differed between the EO-CRC and LO-CRC groups (*p* = 0.023). EO-CRC had fewer surgery-only cases (28.8% vs. 34.3%) and more chemotherapy-only (12.5% vs. 9.3%) and non-surgical chemoradiotherapy (5.8% vs. 1.3%), while the proportion receiving surgery plus chemotherapy was similar (25.0% vs. 25.1%).

Among surgical patients, perioperative treatment timing is shown in Table 5. The proportions of surgery alone, adjuvant-only therapy, and any neoadjuvant therapy were similar between EO-CRC and LO-CRC (*p* = 0.343), although neoadjuvant therapy was more frequent in EO-CRC (11.7% vs. 6.8%).

### 3.5. Molecular Characteristics

The number of patients with available molecular test results varied by marker: MSI (*n* = 530), KRAS (*n* = 699), NRAS (*n* = 673), BRAF (*n* = 127), TP53, and APC (*n* = 81 each). Molecular characteristics are summarized in Table 6. Because molecular testing was performed selectively in routine clinical practice, comparisons for low-frequency markers—particularly BRAF, TP53, and APC—should be interpreted as exploratory.

MSI status was assessed in 43 and 487 patients with EO-CRC and LO-CRC, respectively. MSS was the most common in both groups (88.4% and 84.8%, respectively). MSI-L was observed in 9.3% of EO-CRC and 8.2% of LO-CRC, and MSI-H in 2.3% and 7.0%, respectively; the distribution was not significantly different between groups (*p* = 0.524).

KRAS mutations were less frequent in patients with EO-CRC than in those with LO-CRC (32.7% vs. 47.1%); however, this difference did not reach statistical significance (*p* = 0.059). NRAS mutations were uncommon in both groups (4.2% vs. 3.4%, *p* = 1.000). No BRAF mutations were detected in the EO-CRC patients (0/12), whereas 3.5% of the LO-CRC patients (4/115) harbored BRAF mutations (*p* = 1.000). TP53 mutations were more frequent in patients with EO-CRC (55.6% vs. 37.5%), although the difference was not statistically significant (*p* = 0.471). APC was wild-type in all analyzable cases.

### 3.6. Survival Outcomes

**Figure 2:**
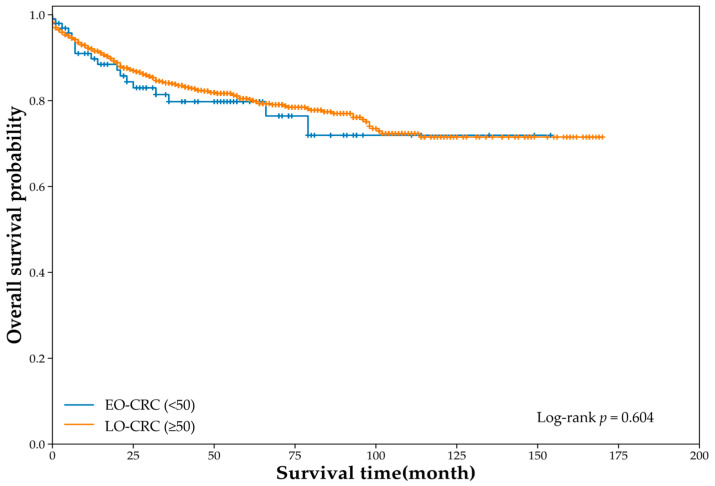
Overall survival according to age group (unadjusted Cox: HR 1.14, 95% CI 0.70–1.84).

For the OS analysis, 1366 patients were included (EO-CRC, *n* = 103; LO-CRC, *n* = 1263). During the follow-up period, death occurred in 18 patients with EO-CRC and 197 patients with LO-CRC. Kaplan–Meier curves demonstrated no significant difference in OS between groups (log-rank χ^2^ = 0.269, *p* = 0.604; Figure 2). In univariate Cox regression, EO-CRC was not associated with OS compared with LO-CRC (HR 1.14, 95% CI 0.70–1.84).

**Figure 3:**
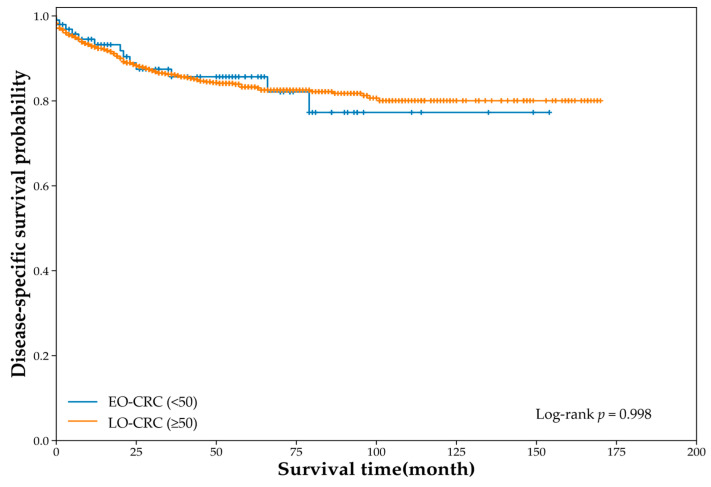
Disease-specific survival according to age group (unadjusted Cox: HR 1.00, 95% CI 0.57–1.76).

For the DSS analysis, 1366 patients were included (EO-CRC, *n* = 103; LO-CRC, *n* = 1263). During the follow-up period, CRC-related deaths occurred in 13 patients with EO-CRC and 159 patients with LO-CRC. DSS curves were comparable between groups, with no significant difference by log-rank test (log-rank χ^2^ = 0.000006, *p* = 0.998; Figure 3). The univariate Cox model similarly showed no difference (HR 1.00, 95% CI 0.57–1.76).

**Figure 4:**
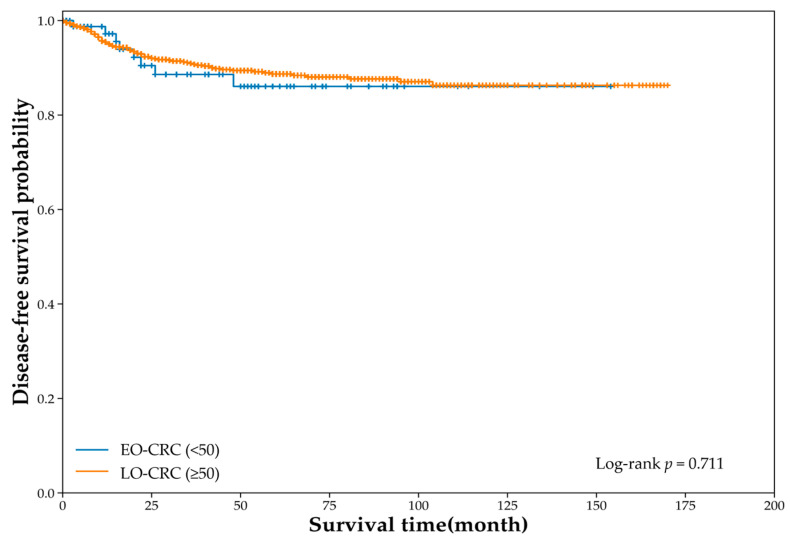
Disease-free survival according to age group (unadjusted Cox: HR 1.15, 95% CI 0.56–2.36).

For the DFS analysis, 1342 patients were included (EO-CRC, *n* = 93; LO-CRC, *n* = 1249). During the follow-up period, recurrence events occurred in 8 patients with EO-CRC and 93 patients with LO-CRC. Kaplan–Meier analysis demonstrated no significant difference in DFS between groups (log-rank χ^2^ = 0.137, *p* = 0.711; Figure 4). Univariate Cox regression also showed no significant association between age group and recurrence risk (HR 1.15, 95% CI 0.56–2.36).

### 3.7. Multivariate Analysis for OS

The results of the multivariable Cox proportional hazards model for OS are presented in Table 7. Among 1339 patients without missing covariates (213 deaths), EO-CRC was not independently associated with OS compared with LO-CRC (HR 1.36, 95% CI: 0.80–2.29; *p* = 0.253). Comorbidity showed a borderline association with mortality (HR 1.34, 95% CI: 0.99–1.80; *p* = 0.056), whereas smoking and alcohol consumption were not independently associated with OS. Higher serum albumin levels were independently associated with lower mortality (per 1 g/dL increase: HR 0.45, 95% CI: 0.35–0.57; *p* < 0.001). Tumor location was associated with survival, with right-sided tumors showing higher mortality than left-sided tumors (HR 1.57, 95% CI: 1.13–2.17; *p* = 0.007), while rectal location was not significant. Compared with AJCC stage I, stage III showed a trend toward higher mortality (HR 1.74, 95% CI: 0.92–3.29; *p* = 0.087), and stage IV was strongly associated with worse survival (HR 7.17, 95% CI: 3.84–13.38; *p* < 0.001). In terms of treatment, chemotherapy only (HR 4.44, 95% CI: 2.61–7.57; *p* < 0.001) and radiotherapy without surgery (HR 3.90, 95% CI: 1.68–9.07; *p* = 0.002) were associated with higher mortality compared with surgery alone, whereas neoadjuvant or adjuvant therapy was not independently associated with OS. Diagnosis period was not significantly associated with survival.

### 3.8. Sensitivity Analyses for Overall Survival

Sensitivity analyses were conducted to evaluate the robustness of the association between age group and overall survival (Table 8). In the primary multivariable model including the entire cohort, EO-CRC was not independently associated with OS compared with LO-CRC (HR 1.36, 95% CI 0.80–2.29; *p* = 0.253). This finding remained consistent after excluding rectal cancer cases (colon cancer only: HR 1.39, 95% CI 0.70–2.76; *p* = 0.345), when stratified by stage (stage I–III: HR 0.76, 95% CI 0.26–2.21; *p* = 0.612; stage IV: HR 1.80, 95% CI 0.96–3.35; *p* = 0.066), and across diagnosis periods (2011–2014: HR 1.22, 95% CI 0.22–6.81; *p* = 0.818; 2015–2018: HR 0.98, 95% CI 0.43–2.21; *p* = 0.955; 2019–2024: HR 1.64, 95% CI 0.74–3.67; *p* = 0.225), with no statistically significant age-related difference in survival observed in any subgroup.

## 4. Discussion

This single-center retrospective cohort study compared the clinicopathological and molecular genetic characteristics of EO-CRC (<50 years) and LO-CRC (≥50 years) within a tertiary referral cohort, and evaluated survival outcomes (OS, DSS, and DFS). Patients with EO-CRC had higher proportions of current smoking and alcohol consumption and fewer comorbidities than those with LO-CRC; however, the body mass indices were similar (Table 1). Notably, serum albumin levels were significantly higher in patients with EO-CRC, whereas other laboratory parameters and tumor markers did not differ (Table 2). EO-CRC showed a distal predominance, particularly in the rectum, and a relatively earlier depth of invasion and T-stage distribution, while the N stage, M stage, and proportion of stage IV disease were similar between the groups (Table 3). Molecular characteristics were largely comparable (Table 6). Similarly, survival outcomes did not differ significantly between groups (Figure 2, Figure 3 and Figure 4).

Lifestyle-related factors have been repeatedly emphasized as important risk factors for EO-CRC. In meta-analyses, both smoking and alcohol consumption were consistently associated with increased EO-CRC risk [17,18]. In this study, current smoking and drinking habits were more common in patients with EO-CRC than in those with LO-CRC, suggesting that lifestyle exposure profiles may differ by age group, even within a single-center cohort. However, quantitative exposure information (including pack-years, alcohol amount, and drinking patterns, such as binge drinking) was not available; thus, future studies should incorporate exposure intensity.

Albumin is not only a nutritional marker but also a negative acute-phase reactant that reflects systemic inflammation and chronic disease burden, and it has been used as a prognostic indicator in patients with CRC [19,20,21]. Previous comparative studies have reported differences in albumin levels across age groups [22]. In this context, the lower albumin levels observed in older patients may reflect less favorable baseline systemic conditions at diagnosis, underscoring the importance of a comprehensive pretreatment assessment—including nutritional status, functional status, and comorbidities—in LO-CRC.

CEA and CA19-9 levels did not differ significantly between the EO-CRC and LO-CRC groups, aligning with the clinical understanding that these markers have limited sensitivity for CRC screening and are mainly used as adjunctive tools for post-treatment surveillance and recurrence monitoring [23,24].

EO-CRC has been repeatedly reported to occur more frequently in the distal colorectum, particularly in the rectum, in both Asian and Western studies [15,25]. The distal predominance observed in our cohort suggests that symptoms such as rectal bleeding or changes in bowel habits in younger adults may be attributed to benign conditions, potentially delaying diagnosis [26]. Thus, lowering barriers to endoscopic evaluation in patients < 50 years of age who present with alarming symptoms and risk factors may be a practical strategy.

Although EO-CRC is often described as presenting at more advanced stages because it falls outside routine screening age ranges, real-world data are inconsistent [27,28]. In our cohort, EO-CRC showed an earlier depth of invasion and T stage distribution, yet the proportion of stage IV disease was similar between groups. As stage IV disease reflects the presence of distant metastasis (M1) irrespective of T category [16], similar M1 rates can coexist with different T-stage patterns. In stratified analyses, the earlier T-stage distribution in EO-CRC was largely confined to M0 disease, whereas metastatic tumors were predominantly T4 in both age groups (Appendix A Table A2). This pattern may, in part, reflect differences in diagnostic pathways and endoscopic access, particularly in healthcare systems such as Korea’s, where colonoscopy is widely available beyond the organized screening program [29].

Higher frequencies of poorly differentiated, mucinous, or signet ring cell carcinomas have been reported in EO-CRC, and a recent meta-analysis suggested an increased frequency of high-grade and mucinous/signet ring histology in EO-CRC [26,30]. In contrast, no significant differences were observed in histological grades or subtypes. This may be due to the low absolute frequency of these subtypes (limiting statistical power).

Treatment patterns differed between EO-CRC and LO-CRC (Table 4). EO-CRC was less often treated with surgery alone and more frequently received non-surgical chemotherapy or chemoradiotherapy, whereas the proportion treated with surgery plus chemotherapy was similar. Previous population-based studies have likewise reported more intensive use of systemic therapy and radiotherapy in younger patients [31,32,33]. In our cohort, the distal predominance of EO-CRC, and particularly rectal involvement, may partly account for the greater use of radiotherapy-based approaches, which are integral to contemporary rectal cancer management [34].

Among surgical patients, perioperative treatment timing did not differ significantly between age groups (Table 5). This suggests that, once resection was pursued, treatment sequencing was primarily determined by tumor-related factors rather than age. The higher proportion of chemoradiotherapy without surgery may reflect nonoperative organ-preservation strategies or non-curative treatment; however, treatment intent could not be fully determined in this retrospective analysis [34,35].

Meta-analyses have suggested that EO-CRC harbors KRAS, BRAF, APC, and NRAS mutations less frequently and TP53 or PTEN mutations more frequently than LO-CRC [36]. In our cohort, KRAS mutations and MSI-H tumors were less frequent in patients with EO-CRC; however, the differences were not significant. Notably, a selection bias may have occurred because molecular testing was performed selectively based on clinical availability rather than uniformly across all patients. In addition, MSI-H is more common in proximal colon cancer; therefore, the MSI distribution may have been influenced by the distal predominance of EO-CRC and the higher proportion of right-sided tumors in patients with LO-CRC [37,38]. These findings should be re-evaluated in large multicenter cohorts with standardized, universal MSI/MMR testing and harmonized NGS panels.

Interpretation of the molecular comparisons in this study warrants caution. Molecular testing was performed as part of routine clinical care and was therefore incomplete and heterogeneous over the study period, with BRAF testing available for only 12 EO-CRC cases and TP53 and APC testing for only 9 EO-CRC cases. As summarized in Table A1, molecular testing became substantially more frequent in the later study period, and targeted NGS was mainly performed in selected patients, particularly those with advanced-stage disease. Consequently, the tested subsets—especially for BRAF and TP53/APC—were enriched for stage IV cases, which may have biased mutation frequency estimates and contributed to the underrepresentation of MSI-H or BRAF-mutant tumors in the overall cohort. In addition, TP53 and APC results were derived from heterogeneous targeted NGS panels, and the absence of APC mutations in the analyzable subset should be interpreted with caution. Accordingly, the molecular comparisons in this study should be regarded as exploratory.

The reported prognostic differences between EO-CRC and LO-CRC vary across studies depending on the design and adjustment strategies, and it is difficult to conclude that EO-CRC uniformly has worse outcomes. Some meta-analyses have suggested comparable or even better OS for EO-CRC with similar cancer-specific survival; however, in rectal cancer subgroups, disease-free survival may be worse [39,40]. In our cohort, despite a tendency toward an earlier T stage and a higher proportion of patients with stage I EO-CRC, survival outcomes were similar, probably owing to stage heterogeneity within EO-CRC, increased rectal cancer burden and treatment complexity, limited sample size and event numbers, and potential selection bias from excluding cases with missing vital statuses in the OS analysis. Thus, our findings should be interpreted as showing comparable survival rates between EO-CRC and LO-CRC rather than implying superior or inferior prognoses.

In multivariable Cox regression analysis, EO-CRC was not independently associated with OS compared with LO-CRC, consistent with large cohort studies and meta-analyses reporting comparable survival after adjustment for stage and other prognostic factors [39,41]. Right-sided tumors were associated with worse survival than left-sided tumors, in line with previous reports [42]. AJCC stage was the strongest predictor of OS, and higher serum albumin levels were independently associated with lower mortality, supporting prior evidence linking hypoalbuminemia to adverse CRC outcomes [43]. Overall, these findings indicate that stage, tumor location, and baseline systemic condition are more important determinants of OS than age alone. The association between age group and OS remained unchanged after adjustment for treatment modality and diagnosis period, suggesting that differences in treatment patterns or calendar period did not account for the comparable survival between EO-CRC and LO-CRC. The higher mortality observed in non-surgical treatment categories likely reflects underlying disease severity and treatment intent rather than a direct treatment effect. Consistent results were observed in sensitivity analyses excluding rectal cancer and stratifying by stage and diagnosis period (Table 8).

Despite the growing clinical concerns regarding early-onset colorectal cancer, there are limited data characterizing its clinical behavior and prognosis, particularly outside Western populations. Most prior studies have focused on Western cohorts or have primarily described clinicopathologic characteristics without robust outcome analyses. In this context, the present study provides clinically relevant real-world evidence by directly comparing EO-CRC and LO-CRC within a well-characterized tertiary-center cohort and by evaluating oncologic outcomes as well as baseline characteristics. This approach allows for a more balanced understanding of EO-CRC, not only as a distinct clinical entity at presentation but also in terms of its prognostic implications in routine clinical practice.

This study has several limitations. First, as this was a single-center retrospective cohort without external validation, selection bias cannot be excluded, and the generalizability of the findings may be limited. Second, smoking and alcohol use were captured as categorical variables (never, former, and current) without detailed quantitative exposure information (e.g., pack-years, drinks per week, or binge-drinking patterns), precluding dose–response analyses and leaving the possibility of residual confounding in lifestyle-associated differences between EO-CRC and LO-CRC. Third, the number of events in the EO-CRC group was relatively small, which may have reduced the precision of the survival estimates. Finally, hereditary CRC syndromes were excluded based on documented diagnoses and available genetic testing; however, germline testing was not performed uniformly throughout the study period, particularly in earlier years, and undiagnosed hereditary cases may have been included. Future multicenter studies with harmonized inclusion criteria and standardized molecular testing protocols are warranted.

## 5. Conclusions

In this single-center tertiary-care cohort, EO-CRC demonstrated distinct clinical features, including higher exposure to smoking and alcohol, distal tumor predominance, and earlier depth of invasion, whereas the molecular characteristics and survival outcomes were largely comparable to those of LO-CRC. Age at diagnosis was not an independent prognostic factor, and the disease stage remained the primary determinant of survival. These findings underscore the potential value of personalized diagnostic frameworks that facilitate early detection in individuals aged <50 years and indicate the need for larger multicenter studies with standardized molecular profiling.

## Figures and Tables

**Table 1:** Baseline characteristics of the study population ^1,2^.

		EO-CRC (*n* = 104)	LO-CRC (*n* = 1279)	Overall	*p*-Value
Sex	Male	53 (51.0)	769 (60.1)	822 (59.4)	0.084
	Female	51 (49.0)	510 (39.9)	561 (40.6)	
	Overall	104 (100)	1279 (100)	1383 (100)	
Age at diagnosis, years		43.30 ± 5.64	69.99 ± 10.86	67.98 ± 12.69	<0.001
Height, cm		164.89 ± 7.73	160.37 ± 9.45	160.70 ± 9.41	<0.001
Weight, kg		64.64 ± 13.24	60.07 ± 11.87	60.40 ± 12.03	0.001
Body mass index, kg/m^2^		23.67 ± 3.91	23.26 ± 3.62	23.29 ± 3.64	0.315
Smoking status	Non-smoker	53 (51.0)	794 (62.1)	847 (61.2)	<0.001
	Ex-smoker	13 (12.5)	246 (19.2)	259 (18.7)	
	Current smoker	38 (36.5)	239 (18.7)	277 (20.0)	
	Overall	104 (100)	1279 (100)	1383 (100)	
Alcohol consumption	Non-drinker	41 (39.4)	700 (54.7)	741 (53.6)	0.002
	Ex-drinker	13 (12.5)	180 (14.1)	193 (14.0)	
	Current drinker	50 (48.1)	399 (31.2)	449 (32.5)	
	Overall	104 (100)	1279 (100)	1383 (100)	
Comorbidity	No	89 (85.6)	453 (35.4)	542 (39.2)	<0.001
	Yes	15 (14.4)	826 (64.6)	841 (60.8)	
	Overall	104 (100)	1279 (100)	1383 (100)	
Symptoms at diagnosis	No	27 (26.2)	460 (36.0)	487 (35.2)	0.059
	Yes	76 (73.8)	819 (64.0)	895 (64.8)	
	Overall	103 (100)	1279 (100)	1382 (100)	

^1^ Categorical variables are presented as numbers (%). ^2^ Continuous variables are presented as means ± standard deviations.

**Table 2:** Laboratory findings and tumor markers at diagnosis ^1^.

	EO-CRC	LO-CRC	*p*-Value
Hb (g/dL)	11.99 ± 2.91	11.76 ± 2.53	0.437
WBC (×10^3^/µL)	8.48 ± 4.22	8.19 ± 3.74	0.452
ALB (g/dL)	3.98 ± 0.62	3.84 ± 0.56	0.013
AST (IU/L)	32.31 ± 39.23	30.20 ± 39.82	0.605
ALT (IU/L)	24.82 ± 20.28	20.41 ± 25.14	0.084
CEA (ng/mL)	81.66 ± 244.23	54.40 ± 190.63	0.286
CA19-9 (U/mL)	104.95 ± 324.10	103.28 ± 335.61	0.964

^1^ Continuous variables are presented as mean ± standard deviation.

**Table 3:** Clinicopathologic characteristics ^1^.

		EO-CRC	LO-CRC	Overall	*p*-Value
Tumor location	Rectum	48 (46.2)	322 (25.2)	370 (26.8)	<0.001
	Left	38 (36.5)	622 (48.6)	660 (47.7)	
	Right	18 (17.3)	335 (26.2)	353 (25.5)	
	Overall	104 (100)	1279 (100)	1383 (100)	
Differentiation	Well	10 (10.4)	90 (7.3)	100 (7.5)	0.522
	Moderate	79 (81.2)	1039 (83.8)	1117 (83.6)	
	Poor	8 (8.3)	111 (9.0)	119 (8.9)	
	Overall	96 (100)	1240 (100)	1336 (100)	
Histological subtype	Adenocarcinoma	100 (99.0)	1272 (99.7)	1372 (99.6)	0.101
	Mucinous	0 (0.0)	4 (0.3)	4 (0.3)	
	Signet ring cell	1 (1.0)	0 (0.0)	1 (0.1)	
	Overall	101 (100)	1276 (100)	1377 (100)	
Depth of invasion	Submucosa	22 (31.9)	245 (19.8)	267 (20.4)	<0.001
	Proper muscle	16 (23.2)	95 (7.7)	111 (8.5)	
	Serosa	31 (44.9)	900 (72.6)	931 (71.1)	
	Overall	69 (100)	1240 (100)	1309 (100)	
Lymphatic invasion	No	36 (53.7)	418 (48.3)	454 (48.7)	0.447
	Yes	31 (46.3)	448 (51.7)	479 (51.3)	
	Overall	67 (100)	866 (100)	933 (100)	
Vascular invasion	No	39 (59.1)	463 (53.5)	502 (53.9)	0.443
	Yes	27 (40.9)	402 (46.5)	429 (46.1)	
	Overall	66 (100)	865 (100)	931 (100)	
Perineural invasion	No	37 (61.7)	469 (56.3)	506 (56.7)	0.500
	Yes	23 (38.3)	364 (43.7)	387 (43.3)	
	Overall	60 (100)	833 (100)	893 (100)	
Lymph node metastasis	No	51 (55.4)	628 (50.2)	679 (50.6)	0.388
	Yes	41 (44.6)	622 (49.8)	663 (49.4)	
	Overall	92 (100)	1250 (100)	1342 (100)	
Distant metastasis	No	70 (72.2)	965 (76.6)	1035 (76.3)	0.322
	Yes	27 (27.8)	294 (23.4)	321 (23.7)	
	Overall	97 (100)	1259 (100)	1356 (100)	
T stage	Tis	8 (7.9)	122 (9.6)	130 (9.5)	0.001
	T1	16 (15.8)	132 (10.4)	148 (10.8)	
	T2	17 (16.8)	97 (7.6)	114 (8.3)	
	T3	25 (24.8)	533 (41.8)	558 (40.6)	
	T4	35 (34.7)	390 (30.6)	425 (30.9)	
	Overall	101 (100)	1274 (100)	1375 (100)	
N stage	N0	50 (50.5)	637 (50.0)	687 (50.0)	1.000
	N1	23 (23.2)	293 (23.0)	316 (23.0)	
	N2	26 (26.3)	342 (26.8)	368 (26.8)	
	N3	0 (0.0)	3 (0.2)	3 (0.2)	
	Overall	99 (100)	1275 (100)	1374 (100)	
M stage	M0	74 (74.0)	975 (76.4)	1049 (76.2)	0.625
	M1	26 (26.0)	301 (23.6)	327 (23.8)	
	Overall	100 (100)	1276 (100)	1376 (100)	
Stage ^2^	Stage 0	8 (8.0)	123 (9.6)	131 (9.5)	<0.001
	Stage I	32 (32.0)	199 (15.6)	231 (16.8)	
	Stage IIA-IIC	8 (8.0)	296 (23.2)	304 (22.1)	
	Stage IIIA-IIIC	25 (25.0)	359 (28.8)	384 (27.9)	
	Stage IV	27 (27.0)	298 (23.4)	325 (23.6)	
	Overall	100 (100)	1275 (100)	1375 (100)	

^1^ Categorical variables are presented as numbers (%). ^2^ Pathologic staging was based on the AJCC 8th edition TNM classification.

**Table 4:** Treatment modality distribution ^1^.

Treatment Modality	EO-CRC	LO-CRC	*p*-Value
No recorded oncologic treatment	25 (24.0%)	343 (26.8%)	
Surgery only	30 (28.8%)	439 (34.3%)	
Surgery + Chemotherapy	26 (25.0%)	321 (25.1%)	
Surgery + Chemotherapy + Radiotherapy	4 (3.8%)	30 (2.3%)	
Surgery + Radiotherapy (no chemotherapy)	0 (0.0%)	8 (0.6%)	
Chemotherapy only	13 (12.5%)	119 (9.3%)	
Chemotherapy + Radiotherapy (no surgery)	6 (5.8%)	16 (1.3%)	
Radiotherapy only	0 (0.0%)	3 (0.2%)	
Overall	104 (100)	1279 (100)	0.023

^1^ Categorical variables are presented as numbers (%).

**Table 5:** Perioperative chemotherapy/radiotherapy timing among surgical patients ^1^.

Perioperative Chemotherapy/Radiotherapy Timing (Surgical Subgroup)	EO-CRC (*n* = 60)	LO-CRC (*n* = 798)	*p*-Value
Surgery alone (no Chemotherapy/Radiotherapy)	30 (50.0%)	439 (55.0%)	
Any neoadjuvant (Chemotherapy/Radiotherapy before surgery)	7 (11.7%)	54 (6.8%)	
Adjuvant only (Chemotherapy/Radiotherapy after surgery)	23 (38.3%)	305 (38.2%)	
Overall	60 (100)	798 (100)	0.343

^1^ Categorical variables are presented as numbers (%).

**Table 6:** Molecular characteristics ^1^.

		EO-CRC	LO-CRC	Overall	*p*-Value
MSI	MSS	38/43 (88.4)	413/487 (84.8)	451/530 (85.1)	0.524
	MSI-L	4/43 (9.3)	40/487 (8.2)	44/530 (8.3)	
	MSI-H	1/43 (2.3)	34/487 (7.0)	35/530 (6.6)	
KRAS	Wild type	35/52 (67.3)	342/647 (52.9)	377/699 (53.9)	0.059
	Mutant type	17/52 (32.7)	305/647 (47.1)	322/699 (46.1)	
	Overall	52/52 (100)	647/647 (100)	699/699 (100)	
NRAS	Wild type	46/48 (95.8)	604/625 (96.6)	650/673 (96.6)	1.000
	Mutant type	2/48 (4.2)	21/625 (3.4)	23/673 (3.4)	
	Overall	48/48 (100)	625/625 (100)	673/673 (100)	
BRAF	Wild type	12/12 (100)	111/115 (96.5)	123/127 (96.9)	1.000
	Mutant type	0/12 (0.0)	4/115 (3.5)	4/127 (3.1)	
	Overall	12/12 (100)	115/115 (100)	127/127 (100)	
TP53	Wild type	4/9 (44.4)	45/72 (62.5)	49/81 (60.5)	0.471
	Mutant type	5/9 (55.6)	27/72 (37.5)	32/81 (39.5)	
	Overall	9/9 (100)	72/72 (100)	81/81 (100)	
APC	Wild type	9/9 (100)	72/72 (100)	81/81 (100)	1.000
	Mutant type	0/9 (0.0)	0/72 (0.0)	0/81 (0.0)	
	Overall	9/9 (100)	72/72 (100)	81/81 (100)	

^1^ Categorical variables are presented as *n*/*N* (%), where *N* is the number tested.

**Table 7:** Multivariate Cox model for overall survival ^1^.

Covariate	Level	Adjusted HR (95% CI)	*p*-Value
Age group	EO-CRC (<50 years)	1.36 (0.80–2.29)	0.253
Comorbidity	Yes	1.34 (0.99–1.80)	0.056
Smoking status	Ex-smoker	1.16 (0.77–1.73)	0.479
	Current smoker	0.80 (0.53–1.20)	0.285
Alcohol consumption	Ex-drinker	1.04 (0.67–1.61)	0.878
	Current drinker	0.87 (0.61–1.25)	0.450
Serum albumin (g/dL)	Per 1 g/dL increase	0.45 (0.35–0.57)	<0.001
Tumor location	Rectum	0.94 (0.65–1.36)	0.730
	Right colon	1.57 (1.13–2.17)	0.007
AJCC Stage	Stage 0	0.63 (0.23–1.70)	0.359
	Stage II	1.09 (0.56–2.11)	0.797
	Stage III	1.74 (0.92–3.29)	0.087
	Stage IV	7.17 (3.84–13.38)	<0.001
Treatment	Any neoadjuvant therapy	1.36 (0.62–2.94)	0.442
	Adjuvant only	1.08 (0.66–1.79)	0.749
	Chemotherapy only	4.44 (2.61–7.57)	<0.001
	Any radiotherapy without surgery	3.90 (1.68–9.07)	0.002
	No recorded oncologic treatment	1.68 (0.96–2.93)	0.068
Diagnosis period	2015–2018	1.13 (0.72–1.49)	0.589
	2019–2024	1.18 (0.73–1.92)	0.493

^1^ Reference categories were LO-CRC (≥50 years), no comorbidity, never smoker, never drinker, left colon, AJCC stage I, surgery alone (no chemotherapy or radiotherapy), and diagnosis period 2011–2014.

**Table 8:** Sensitivity Analyses of Overall Survival ^1^.

Subgroup	*n* (Deaths)	Adjusted HR (95% CI) for EO-CRC	*p*-Value
Entire cohort	1339 (213)	1.36 (0.80–2.29)	0.253
Colon cancer only	989 (162)	1.39 (0.70–2.76)	0.345
Stage I-III	900 (85)	0.76 (0.26–2.21)	0.612
Stage IV	319 (122)	1.80 (0.96–3.35)	0.066
Diagnosis period 2011–2014	167 (29)	1.22 (0.22–6.81)	0.818
Diagnosis period 2015–2018	480 (91)	0.98 (0.43–2.21)	0.955
Diagnosis period 2019–2024	692 (93)	1.64 (0.74–3.67)	0.225

^1^ Reference category was LO-CRC (≥50 years). All models were adjusted for comorbidity, smoking status, alcohol consumption, serum albumin, tumor location, AJCC stage, treatment modality, and diagnosis period, except when stratified by the corresponding variable.

## Data Availability

The data presented in this study are available upon reasonable request from the corresponding authors. The data are not publicly available due to privacy and ethical restrictions.

## Abbreviations

The following abbreviations are used in this manuscript:

AJCC	American Joint Committee on Cancer
ALB	Albumin
ALT	Alanine aminotransferase
AST	Aspartate aminotransferase
BMI	Body mass index
CA19-9	Carbohydrate antigen 19-9
CEA	Carcinoembryonic antigen
CI	Confidence interval
CRC	Colorectal cancer
DSS	Disease-specific survival
DFS	Disease-free survival
EO-CRC	Early-onset colorectal cancer
HR	Hazard ratio
IRB	Institutional Review Board
LO-CRC	Late-onset colorectal cancer
MSI	Microsatellite instability
MSI-H	MSI-high
MSI-L	MSI-low
MSS	Microsatellite stable
NGS	Next-generation sequencing
OS	Overall survival
PLT	Platelet count
TNM	Tumor–node–metastasis
WBC	White blood cell count

## Appendix A

**Table A1:** Molecular testing availability by age group and calendar period ^1^.

Marker	Period	EO-CRC	LO-CRC
MSI	Overall (2011–2024) ^2^	43/104 (41.3%)	487/1279 (38.1%)
	2011–2014	1/16 (6.2%)	2/160 (1.2%)
	2015–2018	6/34 (17.6%)	63/453 (13.9%)
	2019–2024	29/44 (65.9%)	402/636 (63.2%)
KRAS	Overall (2011–2024)	52/104 (50.0%)	647/1279 (50.6%)
	2011–2014	1/16 (6.2%)	9/160 (5.6%)
	2015–2018	10/34 (29.4%)	166/453 (36.6%)
	2019–2024	33/44 (75.0%)	450/636 (70.8%)
NRAS	Overall (2011–2024)	48/104 (46.2%)	625/1279 (48.9%)
	2011–2014	0/16 (0.0%)	2/160 (1.2%)
	2015–2018	7/34 (20.6%)	151/453 (33.3%)
	2019–2024	33/44 (75.0%)	450/636 (70.8%)
BRAF	Overall (2011–2024)	12/104 (11.5%)	115/1279 (9.0%)
	2011–2014	0/16 (0.0%)	1/160 (0.6%)
	2015–2018	5/34 (14.7%)	46/453 (10.2%)
	2019–2024	7/44 (15.9%)	67/636 (10.5%)
TP53	Overall (2011–2024)	9/104 (8.7%)	72/1279 (5.6%)
	2011–2014	0/16 (0.0%)	0/160 (0.0%)
	2015–2018	3/34 (8.8%)	21/453 (4.6%)
	2019–2024	6/44 (13.6%)	50/636 (7.9%)
APC	Overall (2011–2024)	9/104 (8.7%)	72/1279 (5.6%)
	2011–2014	0/16 (0.0%)	0/160 (0.0%)
	2015–2018	3/34 (8.8%)	21/453 (4.6%)
	2019–2024	6/44 (13.6%)	50/636 (7.9%)

^1^ Molecular testing availability is presented as *n*/*N* (%), where *n* is the number with an available test result and *N* is the total number of patients in each group and period. ^2^ Overall denominators include the full cohort (EO-CRC, *n* = 104; LO-CRC, *n* = 1279); period-specific denominators exclude patients with missing diagnosis date.

**Table A2:** Distribution of T stage stratified by M stage ^1^.

Age Group	M Stage	Tis	T1	T2	T3	T4	Total
EO-CRC	M0	8 (10.8%)	16 (21.6%	17 (23.0%)	22 (29.7%)	11 (14.9%)	74 (100)
	M1	0 (0.0%)	0 (0.0%)	0 (0.0%)	2 (8.0%)	23 (92.0%)	25 (100)
LO-CRC	M0	121 (12.4%)	129 (13.3%)	96 (9.9%)	476 (48.9%)	151 (15.5%)	973 (100)
	M1	1 (0.3%)	3 (1.0%)	1 (0.3%)	56 (18.7%)	238 (79.6%)	299 (100)

^1^ Values are presented as *n* (% within each M-stage stratum). This table includes only patients with available T and M stage information.
